# Bushen-Yizhi formula ameliorates cognitive dysfunction through SIRT1/ER stress pathway in SAMP8 mice

**DOI:** 10.18632/oncotarget.17638

**Published:** 2017-05-04

**Authors:** Shi-Jie Zhang, Ting-Ting Xu, Lin Li, Yu-Min Xu, Zi-Ling Qu, Xin-Chen Wang, Shui-Qing Huang, Yi Luo, Na-Chuan Luo, Ping Lu, Ya-Fei Shi, Xin Yang, Qi Wang

**Affiliations:** ^1^ Institute of Clinical Pharmacology, Guangzhou University of Chinese Medicine, Guangzhou 510405, China; ^2^ Department of Pharmacy, The Fifth Affiliated Hospital of Guangzhou Medical University, Guangzhou 510700, China

**Keywords:** aging, dementia, Bushen-Yizhi formula, SIRT1, ER stress

## Abstract

The Chinese formula Bushen-Yizhi (BSYZ) has been reported to ameliorate cognitive dysfunction. However the mechanism is still unclear. In this study, we employ an aging model, SAMP8 mice, to explore whether BSYZ could protect dementia through SIRT1/endoplasmic reticulum (ER) stress pathway. Morris water maze and the fearing condition test results show that oral administration of BSYZ (1.46 g/kg/d, 2.92 g/kg/d and 5.84 g/kg/d) and donepezil (3 mg/kg/d) shorten the escape latency, increase the crossing times of the original position of the platform and the time spent in the target quadrant, and increase the freezing time. BSYZ decreases the activity of acetylcholinesterase (AChE), and increases the activity of choline acetyltransferase (ChAT) and the concentration of acetylcholine (Ach) in both hippocampus and cortex. In addition, western blot results (Bcl-2, Bax and Caspase-3) and TUNEL staining show that BSYZ prevents neuron from apoptosis, and elevates the expression of neurotrophic factors, including nerve growth factor (NGF), postsynapticdensity 95 (PSD95) and synaptophysin (SYN), in both hippocampus and cortex. BSYZ also increases the protein expression of SIRT1 and alleviates ER stress-associated proteins (PERK, IRE-1α, eIF-2α, BIP, PDI and CHOP). These results indicate that the neuroprotective mechanism of BSYZ might be related with SIRT1/ER stress pathway.

## INTRODUCTION

Senescence is characterized by a complex process of molecular, cellular, and organ damage [1]. It leads to progressive loss of function and increases susceptibility to death and disease including cardiovascular disease, diabetes, osteoporosis, cancer etc. Aging is a well-established risk factor for dementia [2, 3], which aggravates with increasing age [4]. As reported, the prevalence of dementia is about 1 percent at the age of 60, doubles every 5 years, and reaches 30 to 50 percent by the age of 85 [5]. WHO estimates that the rate of dementia will be doubled every 20 years, and reaching 115.4 million in 2050 [6].

The clinical syndromes of dementia including acquired losses of cognitive, emotional abilities severe enough to interfere with daily functioning and the quality of life [7]. It is a progressive accumulation of damaged molecules (DNA oxidation/mutation, protein modification, protein aggregation, lipid peroxidation) and impaired energy metabolism (calorie intake, insulin resistant, oxyradical production, glycation) in brain cells [8]. As more people are living longer, dementia is becoming more common in the population. However, limited proposed aging interventions have received good press. With the aging population increase, appropriate interventions of dementia are urgently needed.

Bushen-Yizhi formula (BSYZ), a traditional Chinese medicine compound recipe, consists of She Chuang Zi (*Cnidium monnieri* (L.) Cuss., fruit), Ren Shen (*Panax ginseng* C. A. Mey., rhizome), Zhi He Shou Wu (Preparata of *Polygonum multiflorum* Thuna., radix), Mu Dan Pi (*Paeonia suffruticosa* Andr., cortex), Nv Zhen Zi (*Ligustrum lucidum* Ait., fruit) and Gou Qi (*Lycium barbarum* L., fruit) (Ait. Patent no. ZL200610112916.1). In our previous studies, we found that BSYZ could protect against dementia. In a multi-center, randomized, double-blind, controlled clinical trial, patients with dementia show significant increase in mini-mental state examination (MMSE) scores after treatment of BSYZ. In animal experiments, BSYZ could ameliorate the spatial memory and object memory impairments through cholinergic pathways, NGF signaling and anti-apoptosis in ibotenic acid (IBO) induced cognitive dysfunction [9, 10]. However, the neuroprotective mechanism of BSYZ is still obscure.

The unfolded protein response (UPR) is a stress response of the endoplasmic reticulum (ER) to a disturbance in protein folding [11–13]. Aging is recognized as a major modifier of the outcome of ER stress. Accumulated evidences show a shift of the UPR towards its proapoptotic state during aging with decreased or increased expression of the components of the UPR [14–16]. In addition, UPR can be regulated by Sirtuin 1 (SIRT1). Sirt1 reduced ER stress and apoptosis of brown adipocyte *in vivo* and *in vitro* by inhibiting Smad3/ATF4 signal [17]. Sirt1 mitigated miR-204-mediated vascular ER stress to preserve Cav1-dependent endothelial function [18]. SIRT1 belongs to the sirtuin family of nicotinamide adenine dinucleotide (NAD+)-dependent deacetylases, which is involved in aging, stress response, maintenance of genomic integrity, and energy metabolism [19].

In this study, we employed an aging-related model, SAMP8 (senescence-accelerated mouse prone 8) [20], to explore whether BSYZ could improve cognitive dysfunction through ER stress signal. We treated the mice with three different doses (1.46 g/kg/d, 2.92 g/kg/d and 5.84 g/kg/d) of BSYZ. We explore the potential mechanism of BSYZ on ameliorating dementia might be through SIRT1/ER stress signal.

## RESULTS

### BSYZ improves learning and memory of SAMP8 mice

**Figure 1:**
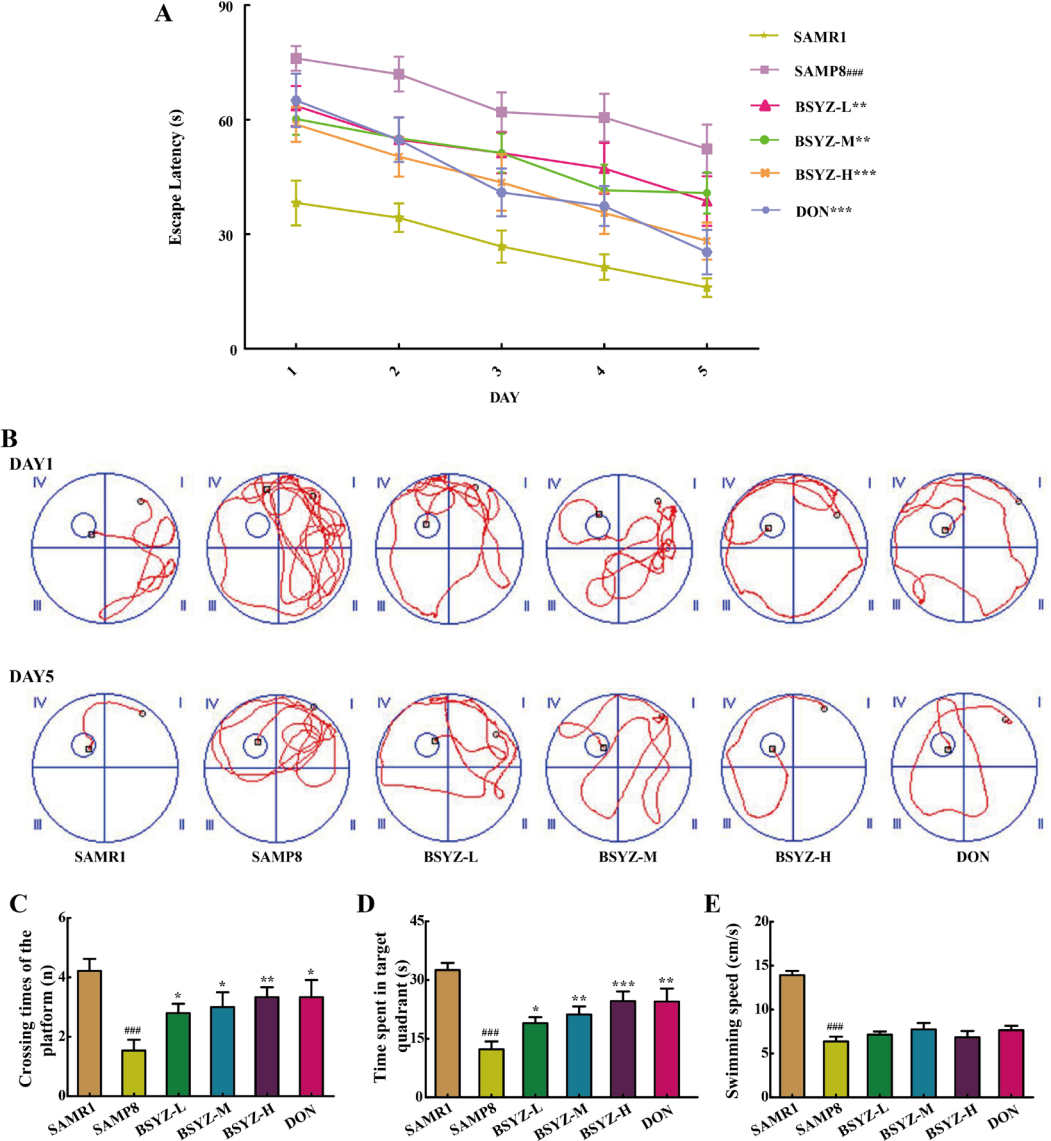
BSYZ ameliorates aging-induced cognitive dysfunction by Morris water maze test in SAMP8 mice (**A**) Escape latency of five consecutive days test. (**B**) The swimming paths of respective groups on the first and fifth day. (**C**) Crossing times of the target platform in the probe trial. (**D**) Time spent in the target quadrant in the probe trial. (**E**) The swimming speed in the probe trial. BSYZ-L: Bushen-Yizhi (1.46 g/kg/d); BSYZ-M: Bushen-Yizhi (2.92 g/kg/d); BSYZ-H: Bushen-Yizhi (5.84 g/kg/d); DON: donepezil. Data represent mean ± SEM (*n* = 20 per group). ^###^*P* < 0.001 vs. SAMR1; *P < 0.05, **P < 0.01, ***P < 0.001 vs. SAMP8.

In Morris water maze test, the time for mice to find the hidden platform was declined progressively during the five training days (Figure 1A and 1B). In contrast to senescence-accelerated resistant mice 1 (SAMR1) group, the period of time to find the hidden platform remarkably increased in senescence-accelerated mouse prone 8 (SAMP8) group. However, Bushen-Yizhi (BSYZ, low-dose, middle-dose and high-dose) and donepezil (DON, a drug for dementia prevention) obviously shortened escape latency when compared with SAMP8 group, especially for high-dose group (*p* < 0.001, Figure 1A). The SAMP8 group presented a chaotic and longer swimming path, which were improved by BSYZ and DON (Figure 1B). On the sixth day, the probe trial was performed by removing the platform and allowing the mice to swim freely to estimate their spatial-working memory (Figure 1C and 1D). SAMP8 group presented a less time spent in target quadrant, fewer times crossing the position of the removed platform (*p* < 0.001), which were ameliorated by BSYZ and DON. The swimming speed of SAMP8 group was significantly decreased (*p* < 0.001) compared with SAMR1 group, but no obvious difference was observed among SAMP8, BSYZ and DON groups (Figure 1E).

**Figure 2:**
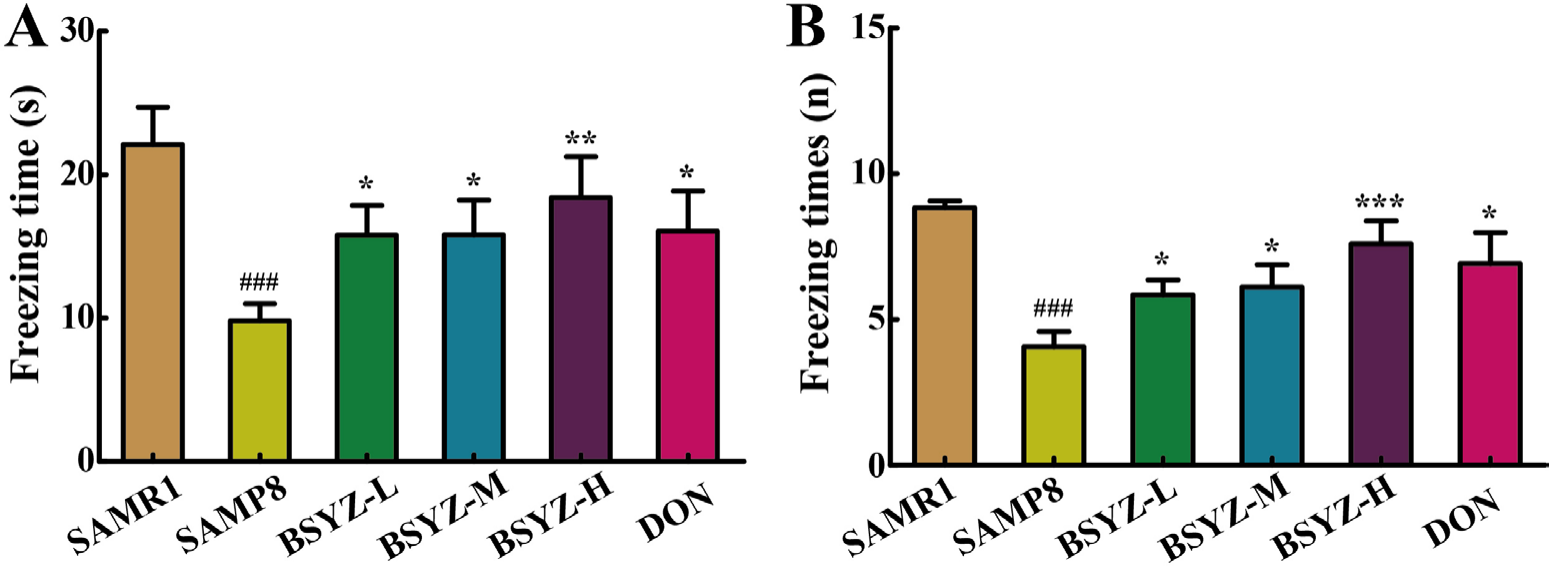
BSYZ ameliorates aging-induced cognitive dysfunction by fearing condition test in SAMP8 mice (**A**) Freezing time of 24 h after anesthesiain FCT. (**B**) Freezing times in fearing condition test (FCT). BSYZ-L: Bushen-Yizhi (1.46 g/kg/d); BSYZ-M: Bushen-Yizhi (2.92 g/kg/d); BSYZ-H: Bushen-Yizhi (5.84 g/kg/d); DON: donepezil. Data represent mean ± SEM (*n* = 20 per group). ^###^*P* < 0.001 vs. SAMR1; *P < 0.05, **P < 0.01, ***P < 0.001 vs. SAMP8.

In fear conditioning test (FCT), differences were observed between SAMR1 and SAMP8 group in both the contextual and the cued recall paradigms. SAMP8 group presented less freezing time and freezing times (*p* < 0.001, Figure 2A and 2B). These were ameliorated after treatment of BSYZ and DON. These results demonstrated that treatment with BSYZ remarkably reversed the cognitive deficits in SAMP8 mice.

### BSYZ improves the cholinergic nerve system in SAMP8 mice

**Figure 3:**
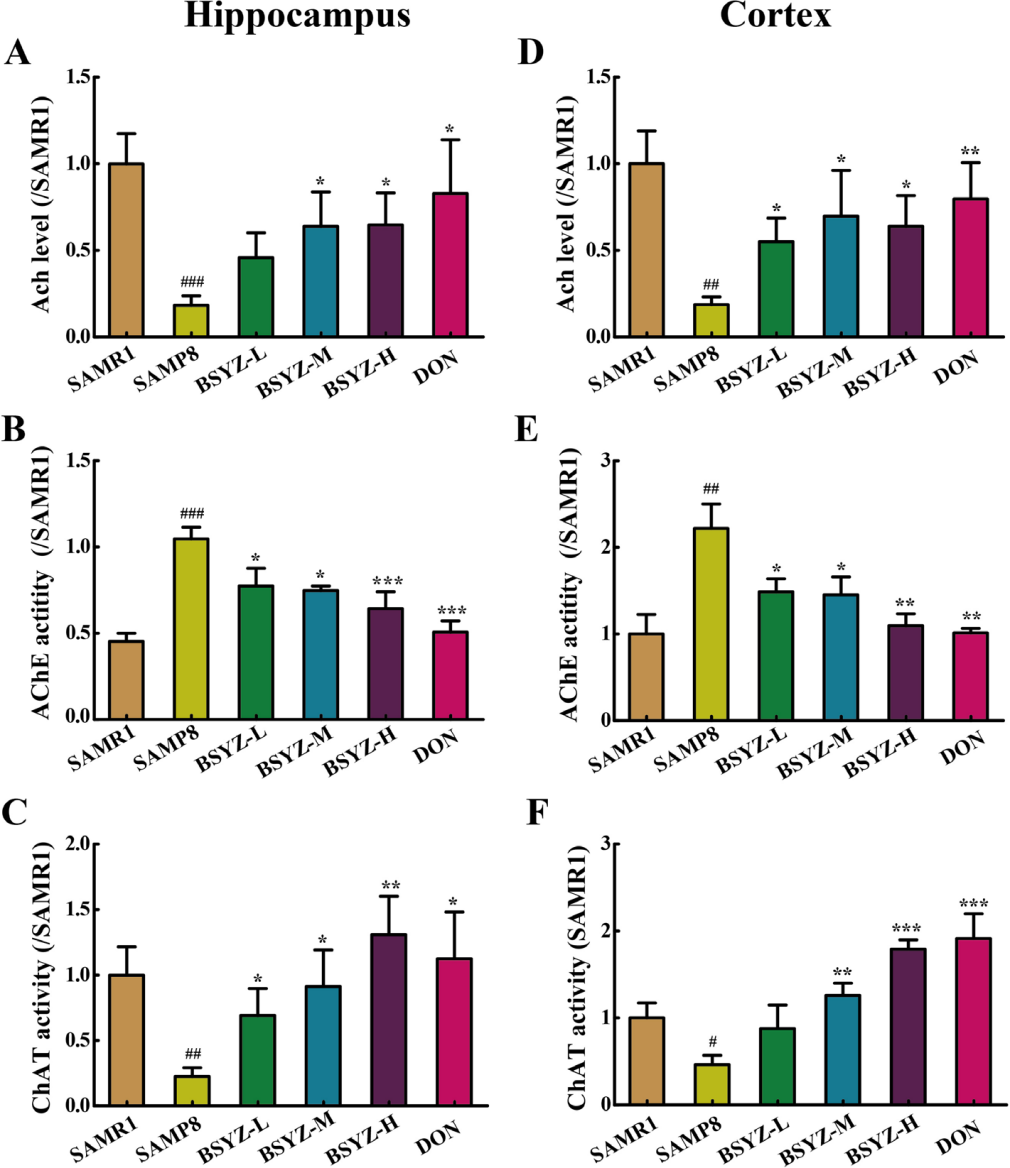
BSYZ improves cholinergic nerve system in SAMP8 mice The supernatant of hippocampus and cortex homogenate was used for the assay. The level of acetylcholine (Ach) and the activities of acetylcholinesterase (AChE) and choline acetyltransferase (ChAT) inhippocampus (**A**, **B** and **C**). The level of Ach and the activities of AChE and ChAT in cortex (**C**, **D**, **E** and **F**). BSYZ-L: Bushen-Yizhi (1.46 g/kg/d); BSYZ-M: Bushen-Yizhi (2.92 g/kg/d); BSYZ-H: Bushen-Yizhi (5.84 g/kg/d); DON: donepezil. Data represent mean ± SEM (*n* = 20 per group). ^#^*P* < 0.05, ^##^*P* < 0.01, ^###^*P* < 0.001 vs. SAMR1; *P < 0.05, **P < 0.01, ***P < 0.001 vs. SAMP8.

To illuminate the potential mechanism of BSYZ in ameliorating cognition deficiency in SAMP8 mice, the activities of cholinergic marker enzymes and the level of acetylcholine (Ach) were detected. A remarkable increase of acetylcholinesterase (AChE) activity in SAMP8 both in hippocampus and cortex (*p* < 0.001, *p* < 0.01, Figure 3B and 3E), while the treatment with BSYZ and DON significantly decreased the AChE activity. The activity of choline acetyltransferase (ChAT) and concentration of Ach in SAMP8 group was decreased sharply in both hippocampus and cortex, whereas BSYZ and DON enhanced the activity of ChAT and increased the concentration of Ach significantly (Figure 3A, 3C, 3D and 3F). Thus, BSYZ could protect cognitive deficits by influencing cholinergic nervous system.

### BSYZ decreases neuronal apoptosis in SAMP8 mice

**Figure 4:**
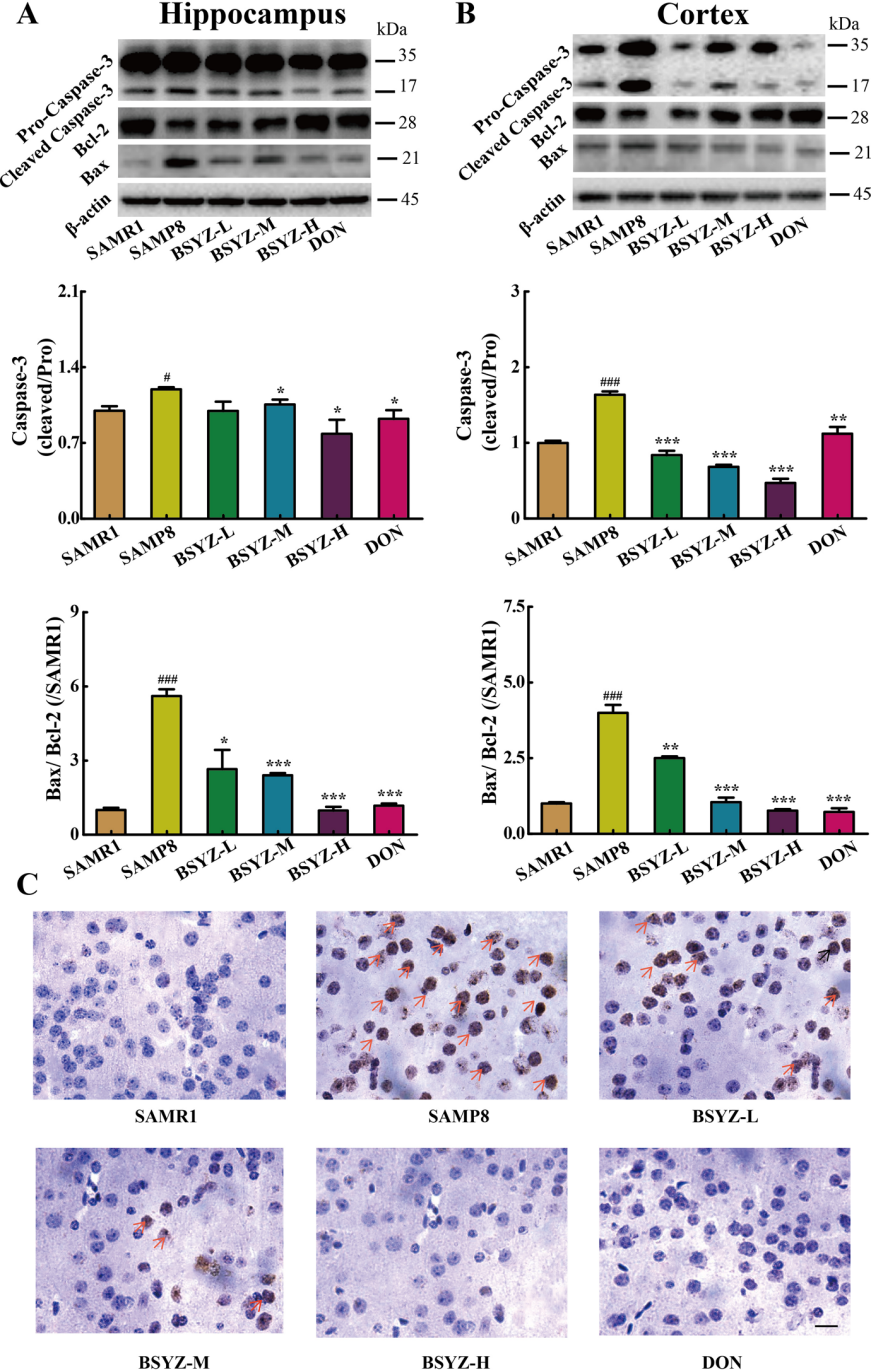
BSYZ protects against apoptosis in both hippocampus and cortex Western blot analysis of Bax, Bcl-2 and Caspase-3 in hippocampus (**A**) and cortex (**B**). (**C**) TUNEL staining in parietal cortex in mice. Scale bar: 50 μm. BSYZ-L: Bushen-Yizhi (1.46 g/kg/d); BSYZ-M: Bushen-Yizhi (2.92 g/kg/d); BSYZ-H: Bushen-Yizhi (5.84 g/kg/d); DON: donepezil. Data represent mean ± SEM (*n* = 20 per group). ^#^*P* < 0.05, ^###^*P* < 0.001 vs. SAMR1; *P < 0.05, **P < 0.01, ***P < 0.001 vs. SAMP8.

As shown in Figure 4A and 4B, the expression of apoptosis-related proteins Bax and cleaved Caspase-3 increased and Bcl-2 decreased in SAMP8 both in hippocampus and cortex. Both BSYZ and DON increased the Bcl-2 expression and decreased the Bax and cleaved Caspase-3 expressions. TUNEL staining was also performed (Figure 4C). TUNEL-positive cells were stained deep brown in the cortex. Compared with SAMR1 mice, TUNEL-positive cells in the cortex of SAMP8 were prominently increased. BSYZ and DON markedly attenuated the neuronal apoptosis in SAMP8 mice. These results demonstrated that BSYZ could improve the ability of anti-apoptosis in SAMP8 mice.

### BSYZ ameliorates neurodegeneration in SAMP8 mice

**Figure 5:**
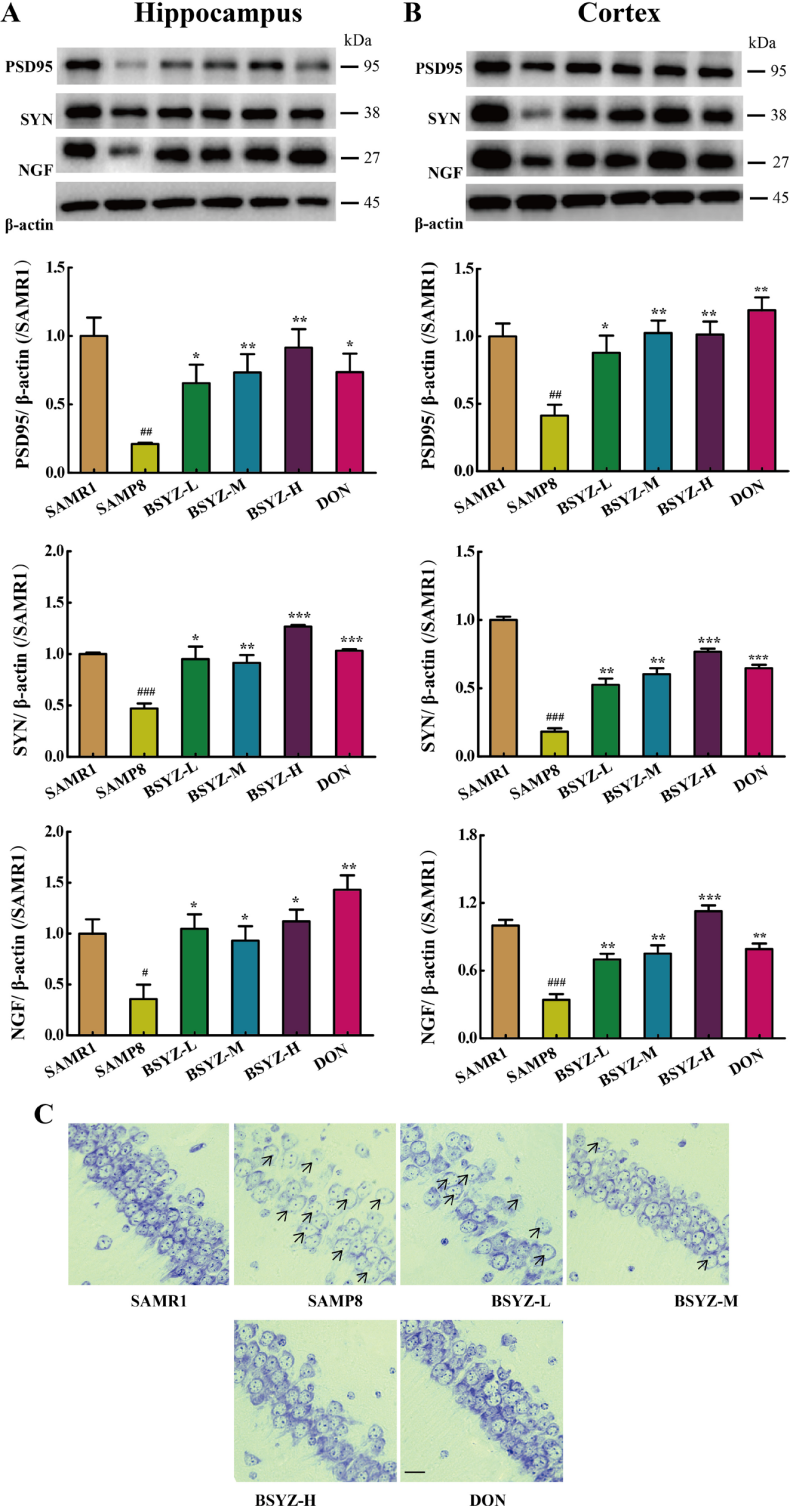
BSYZ ameliorates neurodegeneration in SAMP8 mice Western blot analysis of postsynapticdensity 95 (PSD95), synaptophysin (SYN) and nerve growth factor (NGF) in hippocampus (**A**) and cortex (**B**). (**C**) Nissl's staining in parietal hippocampus. Scale bar: 50 μm. BSYZ-L: Bushen-Yizhi (1.46 g/kg/d); BSYZ-M: Bushen-Yizhi (2.92 g/kg/d); BSYZ-H: Bushen-Yizhi (5.84 g/kg/d); DON: donepezil. Data represent mean ± SEM (*n* = 20 per group). ^#^*P* < 0.05, ^##^*P* < 0.01, ^###^*P* < 0.001 vs. SAMR1; *P < 0.05, **P < 0.01, ***P < 0.001 vs. SAMP8.

As shown in Figure 5, the expression of neurotrophic factors including nerve growth factor (NGF), postsynapticdensity 95 (PSD95) and synaptophysin (SYN) was sharply decreased in SAMP8. While under the treatment of BSYZ and DON, the proteins regain to the normal level (Figure 5A and 5B). Nissl's staining was further detected (Figure 5C). In the hippocampal subfield of SAMP8, the neurons were significantly shrunken, irregularly arranged, and weakly stained, which indicated that neurons were diffusely deteriorated or dead and a great many Nissl bodies lost in these neurons. Significant difference was shown between SAMP8 group and BSYZ group, which displayed regularly arranged, deeply stained and normal form neurons. These results indicated that BSYZ could ameliorate neurodegeneration in SAMP8 mice.

### BSYZ actives SIRT1 and attenuates endoplasmic reticulum (ER) stress in SAMP8 mice

**Figure 6:**
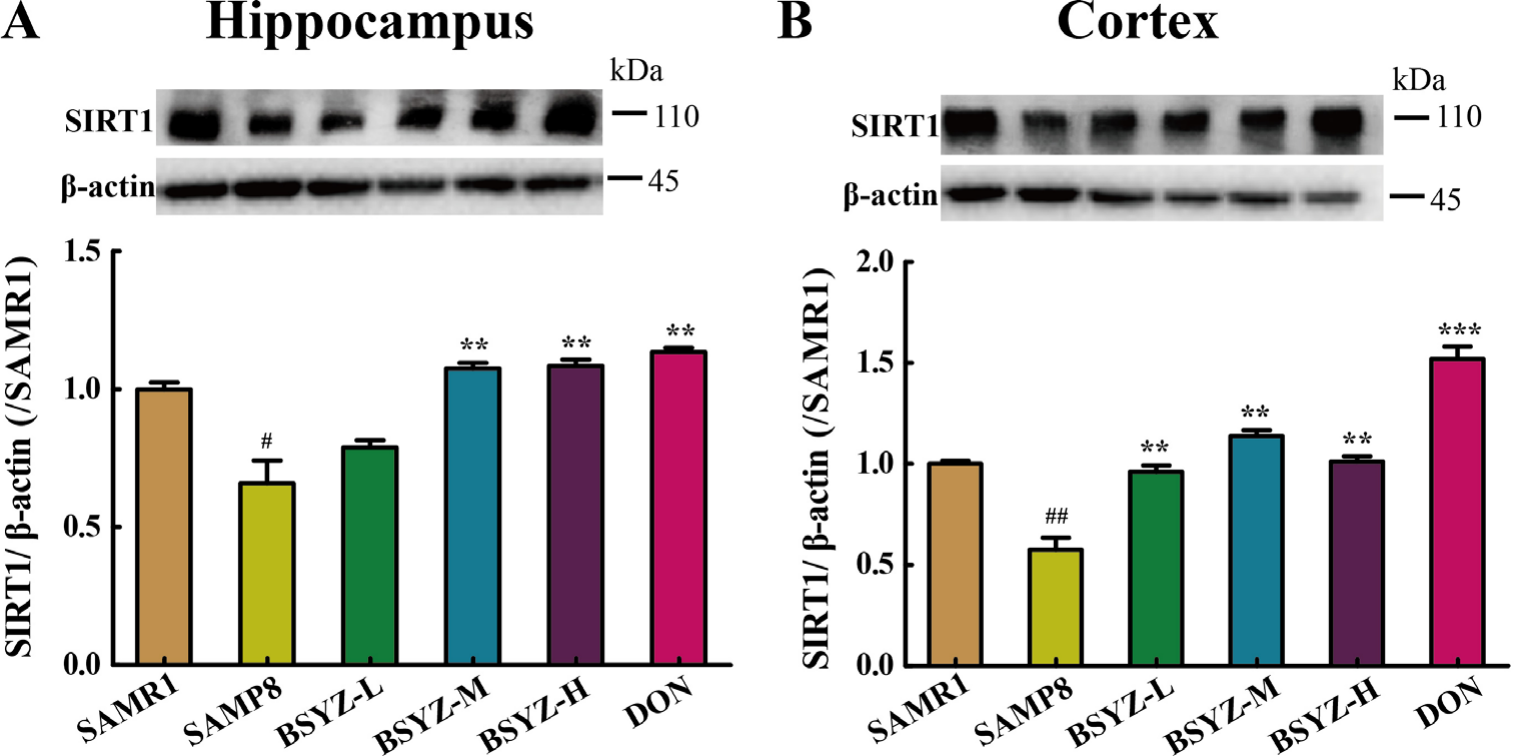
BSYZ up-regulates SIRT1 protein expression in SAMP8 Western blot analysis of the expression of Sirtuin 1 (SIRT1) and quantitative data in hippocampus (**A**) and cortext (**B**). BSYZ-L: Bushen-Yizhi (1.46 g/kg/d); BSYZ-M: Bushen-Yizhi (2.92 g/kg/d); BSYZ-H: Bushen-Yizhi (5.84 g/kg/d); DON: donepezil. Data represent mean ± SEM (*n* = 20 per group). ^#^*P* < 0.05, ^##^*P* < 0.01, vs. SAMR1; **P < 0.01, ***P < 0.001 vs. SAMP8.

**Figure 7:**
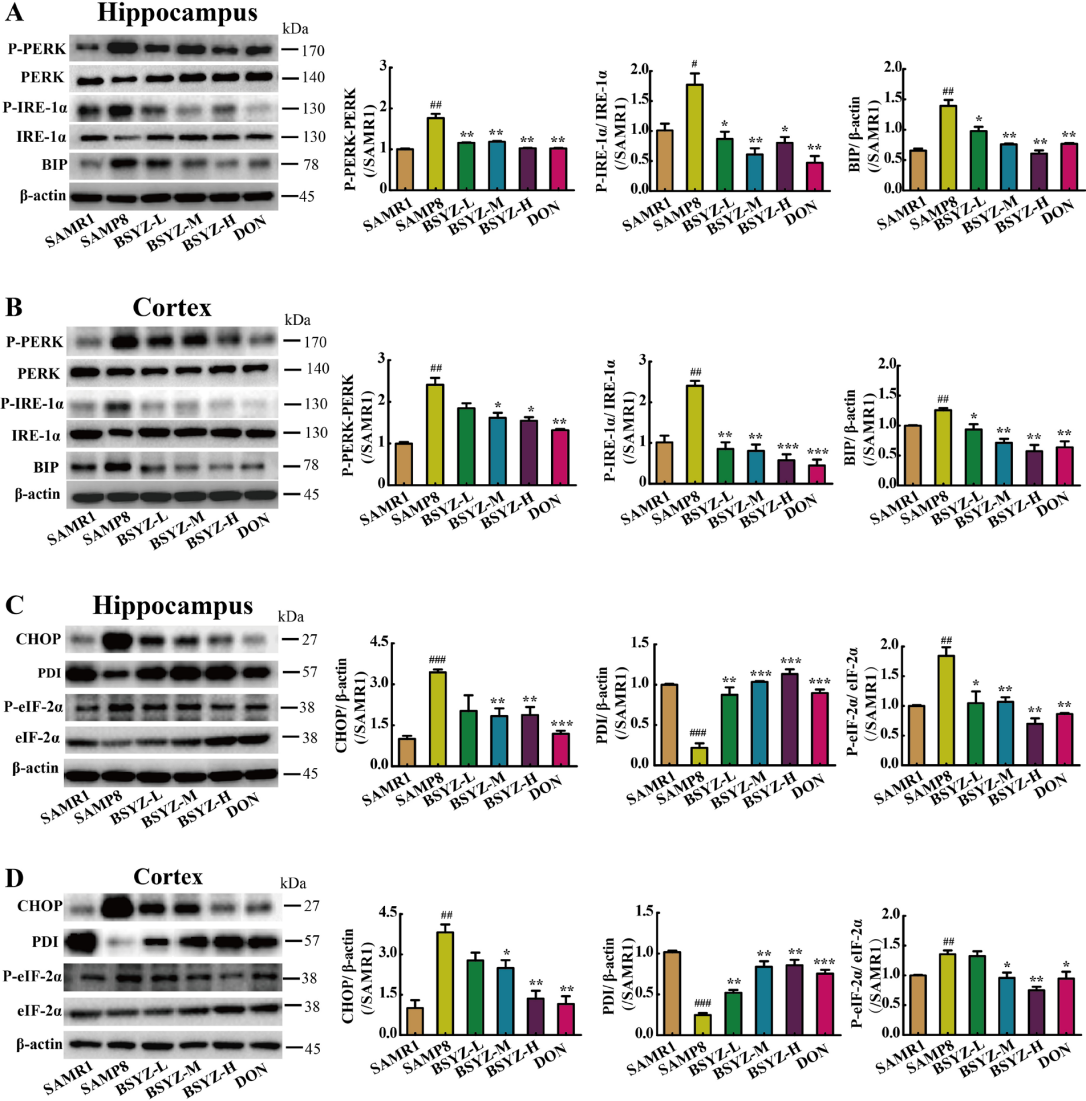
BSYZ ameliorates endoplasmic reticulum (ER) stress in SAMP8 Western blot analysis of PKR-like ER (PERK), phosphorylate PERK (P-PERK), binding immunoglobulin protein (BIP), inositol-requiring enzyme (IRE-1α) and phosphorylate IRE-1α (P- IRE-1α) in hippocampus (**A**) and cortex (**B**). The expression of apoptosis proteinprotein disulfide isomerase (PDI), C/EBP homologous protein (CHOP), initiation factor 2 (eIF-2a) and phosphorylate eIF-2α (P- eIF-2α) in hippocampus (**C**) and cortex (**D**). BSYZ-L: Bushen-Yizhi (1.46 g/kg/d); BSYZ-M: Bushen-Yizhi (2.92 g/kg/d); BSYZ-H: Bushen-Yizhi (5.84 g/kg/d); DON: donepezil. Data represent mean ± SEM (*n* = 20 per group). ^#^*P* < 0.05, ^##^*P* < 0.01, ^###^*P* < 0.001 vs. SAMR1; *P < 0.05, **P < 0.01, ***P < 0.001 vs. SAMP8.

As shown in Figure 6, the expression of SIRT1 decreased in both hippocampus and cortex (*p* < 0.05, *p* < 0.01), but increased in BSYZ (middle- and high-dose) and DON group (*p* < 0.01). Next we measured the protein levels of the two ER stress transducers, PERK and IRE-1α and the expression of chaperones BIP (Figure 7A and 7B). There was an increase in active forms of the effectors of UPR (P-PERK, P-IRE-1α) and BIP in SAMP8 group as compared with the SAMR1, while decreased after the administration of BSYZ. To further evaluate the consequences of ER stress, we next measured the expression levels of CHOP, PDI and eIF-1α. Both CHOP and P-eIF-1α in SAMP8 are up-regulated compared with SAMR1, while down-regulated after the intervention of BSYZ. The expression of PDI sharply decreased but increased under the effect of BSYZ and DON both in hippocampus and cortex (Figure 7C and 7D). These results indicated that the effect of BSYZ on protecting cognitive deficits might be related with SIRT1/ER stress pathway in SAMP8 mice.

## DISCUSSION

In this study, we demonstrate that Chinese formula, BSYZ, is capable of mitigating age-associated cognitive decline in SAMP8 mice. A four-week-administration of BSYZ protected the learning and memory, improved central cholinergic neurotransmission and ameliorated apoptosis in both hippocampus and cortex. In addition, BSYZ activated SIRT1 and attenuated endoplasmic reticulum (ER) stress, which might be an important mechanism of the neuroprotective effect of BSYZ.

Senescence-accelerated mouse prone 8 (SAMP8) inherits early onset and irreversible advancement of senescence based on longevity curves and phenotypic changes characteristic of aging [21]. The mice share similar characteristics with aged humans, such as reduced lifespan, lordosis, hair loss, reduced physical activity [22], elevated biomarkers of oxidative stress [23], inflammation [24], mitochondrial dysfunction [25], brain microvessel defect sand blood-brain barrier dysfunction [26]. The principal phenotypic characteristic is progressive cognitive decline and neurodegenerative changes [27]. Numerous studies have reported that SAMP8 mice have significant memory dysfunction in spatial learning and memory tasks, active and passive avoidance response tasks, fear conditioning and object recognition memory tasks [28]. These features demonstrate that SAMP8 is a successful dementia model. In this study, we found that BSYZ could improve the learning and memory of SAMP8 mice, which is according with our previous studies [9, 10]. Donepezil (DON) showed the similar effect on elevating the availability of ACh in the brain, which has been documented as critical for the effective management of Alzheimer's disease and used as a positive control in ameliorating memory and cognitive impairments [29, 30].

Choline acetyltransferase (ChAT) is a presynaptic cholinergic enzyme, which synthesizes the neurotransmitter acetylcholine (Ach). ChAT activity has been found to be reduced in neurodegenerative disease disease [31, 32]. The neurotransmitter Ach is released from the presynaptic neuron into the synaptic cleft. Then Ach binds to Ach receptors on the postsynaptic membrane, relaying the signal from the nerve [33]. Acetylcholinesterase (AChE), also located on the postsynaptic membrane, terminates the signal transmission by hydrolyzing Ach [33, 34]. Our previous studies have shown that BSYZ could modulate cholinergic system in ibotenic acid (IBO)-treated rat and SCOP-treated mice [10, 35]. In this study, we also detect the cholinergic system in SAMP8 mice. Results proved that BSYZ could regulate cholinergic system. The level of Ach and the activity of ChAT increased and the activity of AChE decreased in both cortex and hippocampus in BSYZ groups compared with SAMP8 group.

Synapses, established by presynaptic axonal terminals and postsynaptic dendritic spines [36, 37], are the specialized intercellular junctions that mediate the transmission of information between neurons. Many studies have reported that the impairment of cognitive ability, learning and memory, is associated with alternation in synaptic [38, 39]. Postsynapticdensity 95 (PSD95) and synaptophysin (SYN) reflect the distribution and density of synapses, which are closely associated with synaptic remodeling. Nerve growth factor (NGF) is required for synaptogenesis during neural development. In our study, neurons were found to be impaired. After the treatment of BSYZ and DON, the low expression of PSD95, SYN and NGF was recovered. Meanwhile, Nissl's staining also proved the neuroprotective effect of BSYZ. These results demonstrated the neuroprotective effect of BSYZ.

SIRT1 has been directly implicated in neuronal protection against stress in cultured cells [40]. In mice, SIRT1 has been shown to protect against neurodegeneration in the p25 over expression model [41], as well as in Wallerian degeneration slow mice [42]. In this study, a down-expressed SIRT1 was found in SAMP8 mice brain, while BSYZ up-regulated this. Some studies indicate that SIRT1 participates in the ER stress response related to inadequate nutrients uptake and its activation protects cardiomyocytes from ER stress-induced cell death [43]. Activation of SIRT1 in the liver of both diet- and obesity-induced T2DM mice strongly attenuated UPR activation by decreasing eIF2-α phosphorylation, XBP-1splicing, and CHOP expression [44]. A SIRT1 activator, resveratrol, could significantly restore cardiac function, reduce cardiomyocyte apoptosis, and ameliorate ER stress [45]. In addition, the activation of SIRT1 decreased acetylation of heat-shock factor protein 1 (HSF1) to modulate the ER stress in N1E115 dopaminergic cells [46]. SIRT1 attenuates endoplasmic reticulum stress induced by palmitate in HepG2 cells via induction of oxygen-regulated protein 150 (ORP150) [47]. Activation of SIRT1 can protect cardiac cells from ER stress through eukaryotic initiation factor 2 (eIF-2a) deacetylation [48].

The endoplasmic reticulum (ER) is a major cellular organelle of the biosynthesis of proteins and the transport of synthesized proteins [12, 13]. Several studies have linked ER function and its stress response pathways to aging [12, 49]. ER stress and activation of the UPR has been implicated in abnormal protein processing and neuronal death in cognitive dysfunction [50]. The unfolded protein response (UPR) is an adaptive cellular response to the disturbance of normal ER functions that attenuates the aggregation of unfolded or misfolded proteins and promotes cell survival [12, 51, 52]. There are three branches of the UPR that are initiated by distinct ER stress sensors located on the ER membrane: inositol-requiring enzyme (IRE-1α) [12], PKR-like endoplasmic reticulum kinase (PERK) and activating transcription factor 6 (ATF6) [12, 53]. Increased P-PERK and P-eIF2α have been reported in postmortem analyses of brains from patients with neurodegenerative diseases [54–57]. When prolonged or overwhelming ER stress, the UPR fails to restore ER homoeostasis, then the apoptotic cascade is activated [58, 59]. In this study, high expressions of phosphorylate PERK (P-PERK) and phosphorylate IRE-1α (P-IRE-1α) in SAMP8 group indicate the occurence of ER stress, accompanied with an increase of chaperones binding immunoglobulin protein (BIP). BSYZ and DON group decrease the expressions to attenuate ER stress. PERK directly phosphorylates the subunit of eukaryotic initiation factor 2 (eIF-2a), which attenuates global mRNA translation to protect cells from ER stress-mediated apoptosis at the initial phase of the UPR [51, 60] and the downstream protein C/EBP homologous protein (CHOP), which indicates the apoptosis. Down-regulated proteins, CHOP and P-eIF-2a, were found in BSYZ and DON groups, which were contrary to SAMP8 group. In addition, the protein disulfide isomerase (PDI), a chaperone, is a part of the quality-control system ensuring correct folding of proteins [61, 62]. The expression of PDI increased under the administration of BSYZ and DON. Bax protein exerts the effect of promoting cell apoptosis, while Bcl-2 present an opposite distinct capacity of anti-apoptosis. Bax activities are regulated though their interaction with Bcl-2 family members. In our study, the apoptotic indexes Bax/Bcl-2 and cleaved Caspase-3 expressions significantly decreased after the treatment of BSYZ both in hippocampus and cortex. Additionally, TUNEL staining showed that BSYZ significantly attenuated the neuronal apoptosis in SAMP8 mice. These results indicated that BSYZ protects against neuronal apoptosis might be related with SIRT1/ER stress pathway.

In this study, we illustrate that BSYZ could ameliorate cognitive dysfunction, which might be related with SIRT1/ ER stress pathway. The further mechanism is still need to study. The Chinese formula, BSYZ, is promised to be a potential anti-dementia drug.

## MATERIALS AND METHODS

### Materials

These traditional Chinese herbal medicines of BSYZ formula were purchased from Guangxi Yifang Chinese Herbal Medicine Department. A voucher specimen (NO.20121209) was deposited at Guangzhou University of Chinese Medicine. The six raw herbs, mixed in the ratio of 3: 3: 2: 2: 2: 2, were extracted and dried to powder. The qualitatively analysis of BSYZ formula had been performed before [35]. Kits used for detection of choline acetyltransferase (ChAT), acetylcholine (Ach) and acetylcholinesterase (AChE) were purchased from the Nanjing Jiancheng Bioengineering Institute (Nanjing, China). Primary antibodies including Sirtuin 1 (SIRT1), PKR-like ER (PERK), phosphorylate PERK (P-PERK), inositol-requiring enzyme (IRE-1α), phosphorylate IRE-1α (P- IRE-1α), eukaryotic initiation factor 2 (eIF-2a), phosphorylate eIF-2α (P- eIF-2α), binding immunoglobulin protein (BIP), protein-disulphide isomerase (PDI), C/EBP homologous protein (CHOP), synaptophysin (SYN), Nerve growth factor (NGF), Postsynapticdensity 95 (PSD95), Bcl-2 and Caspase-3 were purchased from Cell Signaling Technology, Inc. Anti-Bax antibody was purchased from Santa Cruz Biotechnology, Inc. Anti-β-actin was purchased from Sigma-Aldrich. All secondary antibodies (horseradish peroxidase conjugated anti-rabbit IgG and anti-mouse IgG) were purchased from Cell Signaling Technology, Inc. All other reagents were of the highest grade available commercially.

### Animals and treatment

Three-month-old male senescence-accelerated mouse prone 8 (SAMP8) and senescence-accelerated mouse resistant 1 (SAMR1) mice were purchased from the Beijing Vital River Laboratory Animal Technology Co., Ltd. They were all housed and maintained in a specific pathogen-free animal room at 22 ± 2°C with automatic light cycles (12 h light/dark) and a relative humidity of 40–60%. Food and tap water were offered ad libitum throughout the study. The procedures applied in the study were carried out according to the Guiding Principles for the Care and Use of Laboratory Animals that adopted and promulgated by the United States National Institutes of Health. Seven-month-old mice were randomly divided into six groups: vehicle control group (SAMR1, 0.9% saline, *n* = 20), SAMP8 group (*n* = 20), low-dose BSYZ group (SAMP8, BSYZ 1.46 g/kg/d, *n* = 20), middle-dose BSYZ group (SAMP8, BSYZ 2.92 g/kg/d, *n* = 20), high-dose BSYZ group (SAMP8, BSYZ 5.84 g/kg/d, *n* = 20) and donepezil (DON) group (SAMP8, DON 3 mg/kg/d, *n* = 20). Mice were treated with saline, BSYZ and DON, respectively, by gavage, once per day for four weeks.

### The morris water maze test

The Morris water maze test was according to the method of Morris [63]. The water maze equipment (Guangzhou Feidi Biology Technology Co., Ltd., Guangzhou, China) consisted of a black circular pool, a black platform, and a record system. The pool was spatially divided into four imaginary quadrants: target, opposite, left, and right by a computerized tracking/image analyzer system. A circular, transparent escape platform (10 cm diameter) was placed 2 cm below the water surface in the target quadrant of the pool. The learning and memory ability of mice were detected by the Morris water maze test in a dark room. Mice were given a place navigation test for five consecutive days. For each daily trial, there were four sequential training trials beginning with placing the animal in the water facing the wall of the pool with drop location changing for each trial randomly; then the record system starts to record the time. The escape latency was recorded at the end. If the mouse failed to find the platform within 90 s, it would be guided to the platform by the trainer and to remain there for 10 s; its escape latency would be recorded as 90 s. On the sixth day, the mice were allowed to swim freely in the pool for 90 s without the platform. The times of crossing through the original platform position, the time spent in the target quadrant which indicated the degree of memory consolidation and the swimming speed were measured.

### The fear conditioning test

The fear conditioning test (FCT) was performed as those described by Saab et al. with modification [64]. Each mouse was allowed to explore the FCT (Stoelting Co., Wood Dale, IL) chamber equipped with black methacrylate walls, a transparent front door, a speaker and grid floor for 180 s before presentation of a 2-Hz pulsating tone (80 dB, 3,600 Hz) that persisted for 60 s. The tone was followed immediately by a mild foot shock (0.8 mA for 0.5 s). Each mouse was allowed to stay in the chamber for a total of 390 s. Function of learning and memory in the contest test was assessed by measuring the amount of time the mouse demonstrated “freezing behavior”, which is defined as a completely immobile posture except for respiratory efforts during the second 180 s. The second test was performed at 24 h after the anesthesia, respectively.

### Measurement of Ach, AChE and ChAT

All mice were anesthetized and decapitated after behavioral experiments immediately. Hippocampus and cortex were carefully dissected from brains for examination. The hippocampus and cortex were carefully dissected from brains for examination. All the processes were performed on ice-cold plate. Tissues were rapidly stored at – 80°C. The hippocampus and cortex tissues were homogenized with ice-cold saline. The homogenate was centrifuged at 12,000 × g for 10 min at 4°C. The supernatant was collected and the total protein concentration was determined using a bicinchoninic acid (BCA) protein assay kit (Nianjing Jiancheng Bioengineering Institute, Nanjing, China) for the assay of the AChE and ChAT activities and measuring the level of ACh. Then the supernatant was used to detect the Ach concentration and the activities of ChAT and AChE according to the manufacturer's instructions by using Universal Microplate Spectrophotometer (Bio-Rad, Hercules, CA, USA).

### Nissl's staining

Brain paraffin sections were washed in xylene and rehydrated through a graded series of ethanol and double-distilled water. Then the sections were dipped in Nissl's stain (Nanjing Jiancheng Bioengineering Institute, Nanjing, China) for 5–10 min at room tempreture. Then slides were rinsed in double-distilled water and dehydrated through 70%, 95% and 100% alcohol, cleared in xylene. Images were analyzed by using a light microscope and LEICA QWin Plus (Leica Microsystems, Wetzlar, Germany).

### Western blot analysis

The tissues of hippocampus and cortex were homogenized and lysed in sample buffer (0.5 M Tris/HCl pH 6.8, 50% glycerol, 10% sodium dodecyl sulphate (SDS), 1: 100 inhibitor proteases and phosphatases cocktail). The lysate was centrifuged at 12,000 × g for 10 min at 4°C and then denatured by boiling at 100°C with 1: 4 lodding buffer. The same amount of protein (30 μg) was fractionated by SDS-polyacrylamide gel electrophoresis (PAGE) and subsequently transferred onto a polyvinylide fluoride sheets (PVDF) membranes. The membranes were blocked in 5% skim milk that dissolved in Tris-bufferedsaline-Tween-20 (TBST) for 1 h at room temperature. The membranes containing the protein were incubated with anti-SIRT1, anti-PERK, anti-P-PERK, anti-IRE-1α, anti-P-IRE-1α, anti-eIF-2α, anti-p-eIF-2α, anti-BIP, anti-PDI, anti-CHOP, anti-Caspase-3, anti-Bcl-2, anti-SYN, anti-PSD95, anti-Bax, anti-NGF and mouse anti-β-actin overnight at 4°C. Then the membrane was incubated with horseradish peroxidase conjugated anti-rabbit or anti-mouse for 1 h at room temperature. Routinely, protein load was monitored by using a super enhanced chemiluminescence reagent (ECL; Applygen Technologies Inc., Beijing, China).

### TUNEL staining

Brain paraffin sections were washed in xylene and rehydrated through a graded series of ethanol and double distilled water. Then, the sections were washed in PBS and incubated with 50 μl TUNEL reaction mixture for 1 h at 37°C in dark. Further incubation with 50 μl converter-POD was performed at 37°C for 30 min. The sections were then rinsed with PBS and stained with DAB substrate for 10 min at room temperature. TUNEL staining was performed using the *In Situ* Cell Death Detection kit (Roche Diagnostics GmbH, Mannheim, Germany). Images were analyzed by using a light microscope and LEICA QWin Plus (Leica Microsystems, Wetzlar, Germany).

### Statistical analysis

Experimental values were given as means ± S.E.M. All statistical analysis was performed with SPSS 19.0 statistical software (IBM, Endicott, NY). Two-way analysis of variance (ANOVA) was applied to analyze differences in data for the biochemical parameters among the different groups, followed by Dunnett's significant post-hoc test for pair-wise multiple comparisons. The level of statistical significance for all tests was *P* < 0.05.
