# Correction: Bushen-Yizhi formula ameliorates cognitive dysfunction through SIRT1/ER stress pathway in SAMP8 mice

**DOI:** 10.18632/oncotarget.27505

**Published:** 2020-07-07

**Authors:** Shi-Jie Zhang, Ting-Ting Xu, Lin Li, Yu-Min Xu, Zi-Ling Qu, Xin-Chen Wang, Shui-Qing Huang, Yi Luo, Na-Chuan Luo, Ping Lu, Ya-Fei Shi, Xin Yang, Qi Wang

**Affiliations:** ^1^Institute of Clinical Pharmacology, Guangzhou University of Chinese Medicine, Guangzhou 510405, China; ^2^Department of Pharmacy, The Fifth Affiliated Hospital of Guangzhou Medical University, Guangzhou 510700, China

**This article has been corrected:** While reviewing the database for this study, the authors discovered that the Tunel staining data for all 6 panels in Figure 4C did not represent the relevant group. The corrected Figure 4, obtained using original data, is given below. The authors declare that these corrections do not change the results or conclusions of this paper.

Original article: Oncotarget. 2017; 8:49338–49350. 49338-49350. https://doi.org/10.18632/oncotarget.17638

**Figure 4:**
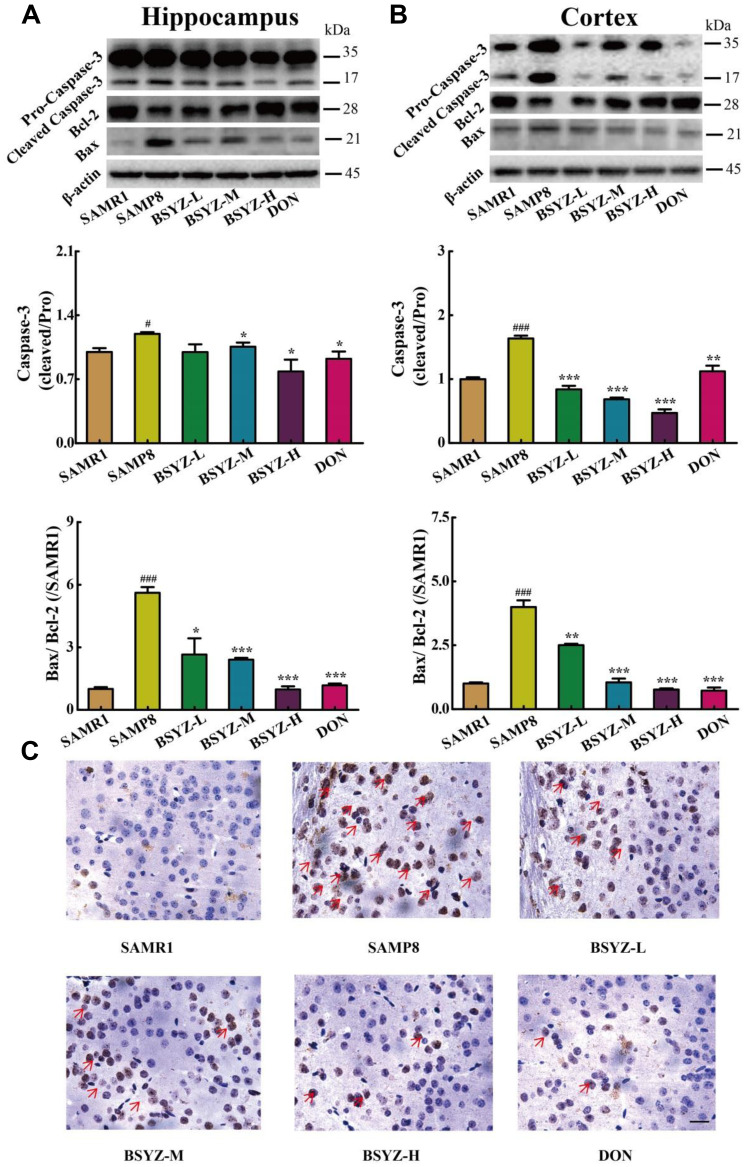
BSYZ protects against apoptosis in both hippocampus and cortex. Western blot analysis of Bax, Bcl-2 and Caspase-3 in hippocampus (**A**) and cortex (**B**). (**C**) TUNEL staining in parietal cortex in mice. Scale bar: 50 μm. BSYZ-L: Bushen-Yizhi (1.46 g/kg/d); BSYZ-M: Bushen-Yizhi (2.92 g/kg/d); BSYZ-H: Bushen-Yizhi (5.84 g/kg/d); DON: donepezil. Data represent mean ± SEM (*n* = 20 per group). ^#^ *P* < 0.05, ^###^ *P* < 0.001 vs. SAMR1; ^*^ *P* < 0.05, ^**^ *P* < 0.01, ^***^ *P* < 0.001 vs. SAMP8.

